# Half metallicity in Cr substituted Fe_2_TiSn

**DOI:** 10.1038/s41598-020-79895-7

**Published:** 2021-01-12

**Authors:** S. Chaudhuri, D. Salas, V. Srihari, E. Welter, I. Karaman, P. A. Bhobe

**Affiliations:** 1 Department of Physics, Indian Institute of Technology Indore, Khandwa Road, Simrol, Indore 453552 India; 2 Department of Materials Science and Engineering, Texas A&M University, College Station, TX 77843 USA; 3 High Pressure and Synchrotron Radiation Physics Division, Bhabha Atomic Research Centre, Mumbai, 400 085 India; 4 Deutsches Elektronen-Synchrotron – A Research Centre of the Helmholtz Association, Notkestraße 85, 22607 Hamburg, Germany

**Keywords:** Spintronics, Electronic properties and materials

## Abstract

Band structure tailoring has been a great avenue to achieve the half-metallic electronic ground state in materials. Applying this approach to the full Heusler alloy Fe_2_TiSn, Cr is introduced systematically at Ti site that conforms to the chemical formula ${\text{Fe}}_{2} {\text{Ti}}_{{1 - x}} {\text{Cr}}_{x}$Sn. Compositions so obtained have been investigated for its electronic, magnetic, and electrical transport properties with an aim to observe the half-metallic ferromagnetic ground state, anticipated theoretically for Fe_2_CrSn. Our experimental study using synchrotron X-ray diffraction reveals that only compositions with $x \le$ 0.25 yield phase pure L2$_1$ cubic structures. The non-magnetic ground state of Fe_2_TiSn gets dramatically affected upon inclusion of Cr giving rise to a localized magnetic moment in the background of Ruderman–Kittel–Kasuya–Yosida (RKKY) correlations. The ferromagnetic interactions begin to dominate for *x* = 0.25 composition. Results of its resistivity and magnetoresistance (MR) measurement point towards a half-metallic ground state. The calculation of exchange coupling parameter, $\hbox {J}_{{ij}}$, and orbital projected density of states that indicate a change in hybridization between 3*d* and 5*p* orbital, support the observations made from the study of local crystal structure made using the extended X-ray absorption fine structure spectroscopy. Our findings here highlight an interesting prospect of finding half-metallicity via band structure tailoring for wide application in spintronics devices.

## Introduction

Modern spintronic industry regards half-metallic materials with 100% spin polarization as the ideal candidates for device fabrication. Such materials display a bandgap at the Fermi level ($\hbox {E}_{{F}}$) in one of the spin bands, while the other spin band conducts the regular metallic character. An early reference to the concept of half-metallicity in Heusler alloys dates back to the extensive work carried out on NiMnSb and related intermetallic compounds^1,2^. Although later findings show some oxides such as $\hbox {CrO}_2$ in thin-film form^3^, or bulk polycrystalline $\hbox {Sr}_{{2}}\hbox {CrReO}_{{6}}$^4^ display the half-metallic electronic structure. Heusler alloys remain the main focus of research in this field due to its ease of tunability, high Curie temperatures and stable crystal structures. Among the full Heusler compositions predicted to be half-metallic, $\hbox {Co}_2$MnSi is the most prominent candidate with Curie temperature as high as 1100 K, and sizeable magnetic moment $\sim$ 6$\mu _{B}$^5^. Other full Heusler alloys such as $\hbox {Mn}_{{2}}$CoAl^6^ and Fe_2_CoSi^7^ are also prominent members of this family of compounds. However, it is essential to note that the applicability of Heuslers is not limited to the field of spintronics alone. These materials are also known for a wide range of other properties like high thermopower generation, magnetic shape memory alloys, and actuators^8^.

Density functional theory(DFT) based first-principles calculations predict that Fe_2_CrSn lies at a crossroad between a barely half-metallic state and a magnetic semiconductor^9^. Such a unique band structure can give rise to fascinating transport and magnetic properties. However, till date, it has not been possible to crystallize pure Fe_2_CrSn in the L2_1_ ordered full Heusler form. On the other hand, Fe_2_TiSn, a non-magnetic semi-metallic alloy, is easy to synthesize and possesses some exciting transport properties^10^. Based on early specific heat measurements it was speculated to be a Kondo insulator^11^, while its low-temperature transport properties match the attributes of a Schottky anomaly^12^. However, the recent investigations explain the transport and magnetic properties of Fe_2_TiSn on the basis of weak localization^13^ and strong spin fluctuation^10^. First principle calculations using generalized gradient approximation(GGA) and density functional theory predicts a half-metallic state and high thermoelectricity in Fe_2_TiSn that could be engineered through proper elemental substitution^14^.

In the quest for half-metallicity in Fe_2_TiSn, a series of compositions with Cr-substitution for Ti bearing chemical formula $\hbox {Fe}_{{2}}\hbox {Ti}_{1-x}\hbox {Cr}_{{x}}$Sn were prepared and investigated. Firstly, the powder diffraction study carried out using synchrotron X-rays confirms that beyond 25% replacement of Ti with Cr gives rise to precipitation of elemental Cr from the composition. Hence the substitution was limited only up to *x* = 0.25. Detailed magnetic measurements reveal the evolution of a magnetically ordered ground state and low-temperature heat capacity studies provide first hints towards the creation of a half-metallic state. The changing hybridization between constituent atoms is evident from the results of extended X-ray absorption fine structure (EXAFS) spectroscopy. Calculation of the Heisenberg exchange coupling parameter further confirms such findings and the orbital projected density of states (DOS) shows the opening of the gap in minority spin density of states. The measurement of electrical resistivity as a function of temperature and applied magnetic field together presents a unified picture of the half-metallic state in $\hbox {Fe}_{{2}}\hbox {Ti}_{0.75}\hbox {Cr}_{0.25}$Sn composition. Very interestingly, the anomalously linear and positive variation of low temperature MR in $\hbox {Fe}_{{2}}\hbox {Ti}_{0.75}\hbox {Cr}_{0.25}$Sn is the notable signature of its half-metallic ground state.

## Results

### X-ray diffraction

**Figure 1:**
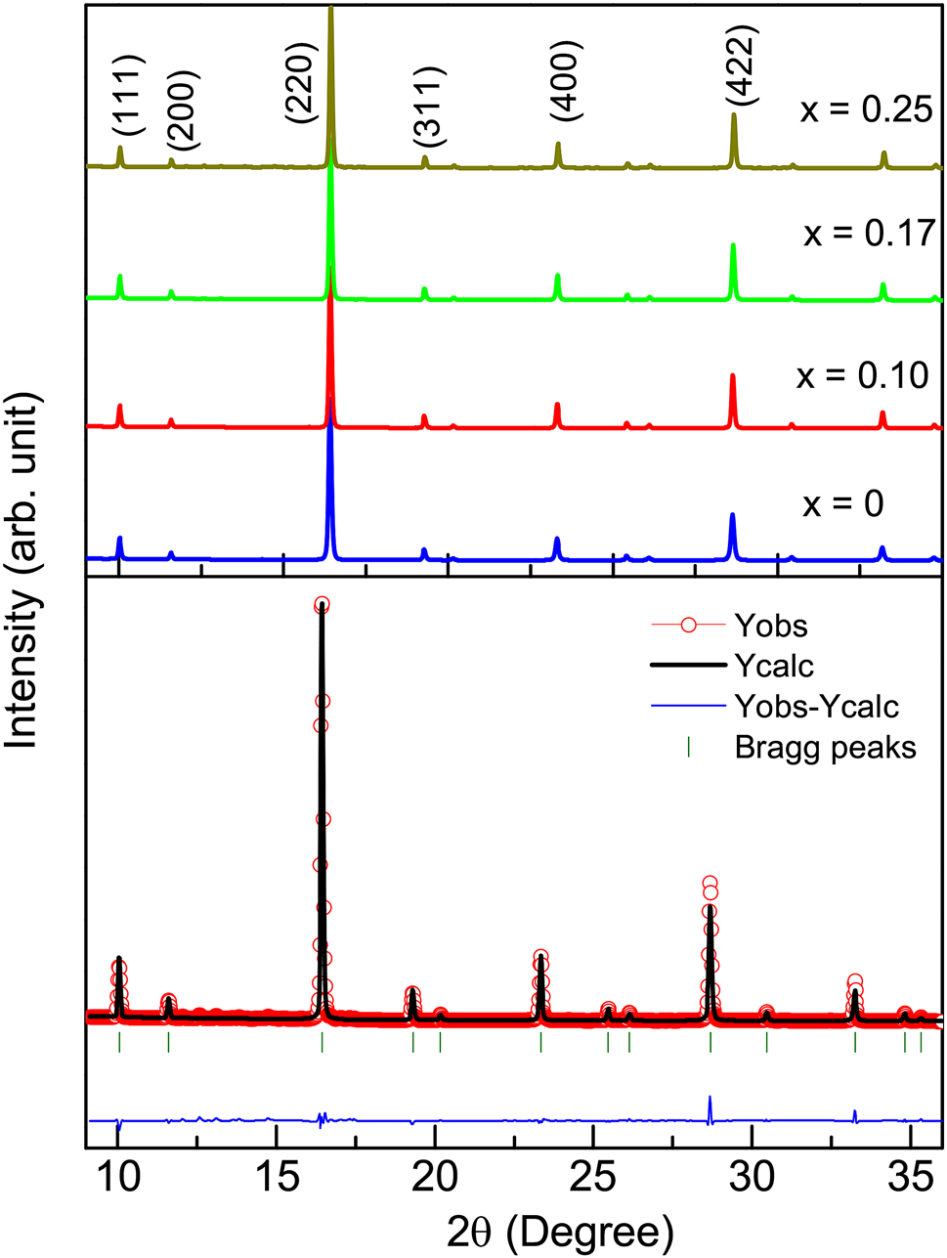
XRD patterns obtained for ${\text{Fe}}_{2} {\text{Ti}}_{{1 - x}} {\text{Cr}}_{x}$Sn compositions using synchrotron source ($\lambda$ = 0.612 Å). The lower panel shows Rietveld refinement of *x* = 0.25 composition.

High intensity synchrotron source X-ray diffraction (XRD) profiles recorded at room temperature for the ${\text{Fe}}_{2} {\text{Ti}}_{{1 - x}} {\text{Cr}}_{x}$Sn (*x* = 0, 0.1, 0.17, 0.25) compositions are shown in Fig. 1. Presence of strong reflections from (111), (200), (220) planes confirm the formation of the expected $\hbox {L2}_{{1}}$ structure with Fm${\bar{3}}$m space group. We find no extra, unaccounted peaks in the profile thus confirming the formation of pure phase compositions. Further, the elemental ratios for each composition obtained from Energy Dispersive X-Ray (EDX) analysis match with the starting stoichiometry (details present in the supplementary text). The recorded XRD profiles were analyzed using Rietveld refinement method as implemented in FullPROF suite^15^. Table 1 summarizes the lattice parameters extracted from the refinement, which shows a linear decrease in lattice constant with the increase in Cr concentration. A decrease in unit cell parameters is due to the smaller size of Cr atoms that replace the Ti atoms.

**Table 1:** Lattice constant (*a*), atomic site-occupancies and parameters determining the goodness-of-fit of the Rietveld refined XRD patterns of the ${\text{Fe}}_{2} {\text{Ti}}_{{1 - x}} {\text{Cr}}_{x}$Sn compositions.

Cr conc.	*a*	site-occupancies				Goodness of fit		
*x*	(Å)	Fe	Ti	Cr	Sn	$\hbox {R}_{{Bragg}}$	$\hbox {R}_{{F}}$	$\chi ^2$
0	6.065 (3)	0.0402	0.0211	–	0.0204	2.17	2.76	2.23
0.10	6.061 (1)	0.0395	0.0196	0.0021	0.0207	3.88	3.36	2.31
0.17	6.058 (1)	0.03981	0.0171	0.0043	0.0208	5.01	5.42	2.12
0.25	6.054 (2)	0.0405	0.0160	0.0059	0.0206	4.12	4.87	2.77

Generally, any multi-component alloy or intermetallic is susceptible to some amount of anti-site disorder between constituent atoms of the composition. The pertinent point here is the sensitivity of physical properties towards such slight structural disorders. The Fe, Ti, and Cr atoms have similar X-ray scattering amplitudes hence relative intensities of (111), (200) super-lattice reflections and refinement of atomic site-occupancies alone, cannot be a comprehensive measure for the L2_1_ ordered state. Rather, the extent of anti-site disorder is judged from the impact it has on the disruption of electronic and structural properties of these compositions. Precise crystal structure investigation determines a small percentage (~ 5%) of inherent Fe/Ti anti-site disorder in Fe_2_TiSn that adversely impacts its low-temperature transport and magnetic properties^10,16^. While the extent of such Fe/Ti disorder in Fe_2_TiSn is in itself small, its relative extent in Cr-substituted compositions reduces further, as will be evident from the experimental findings discussed in the subsequent text.

### Magnetic properties

**Figure 2:**
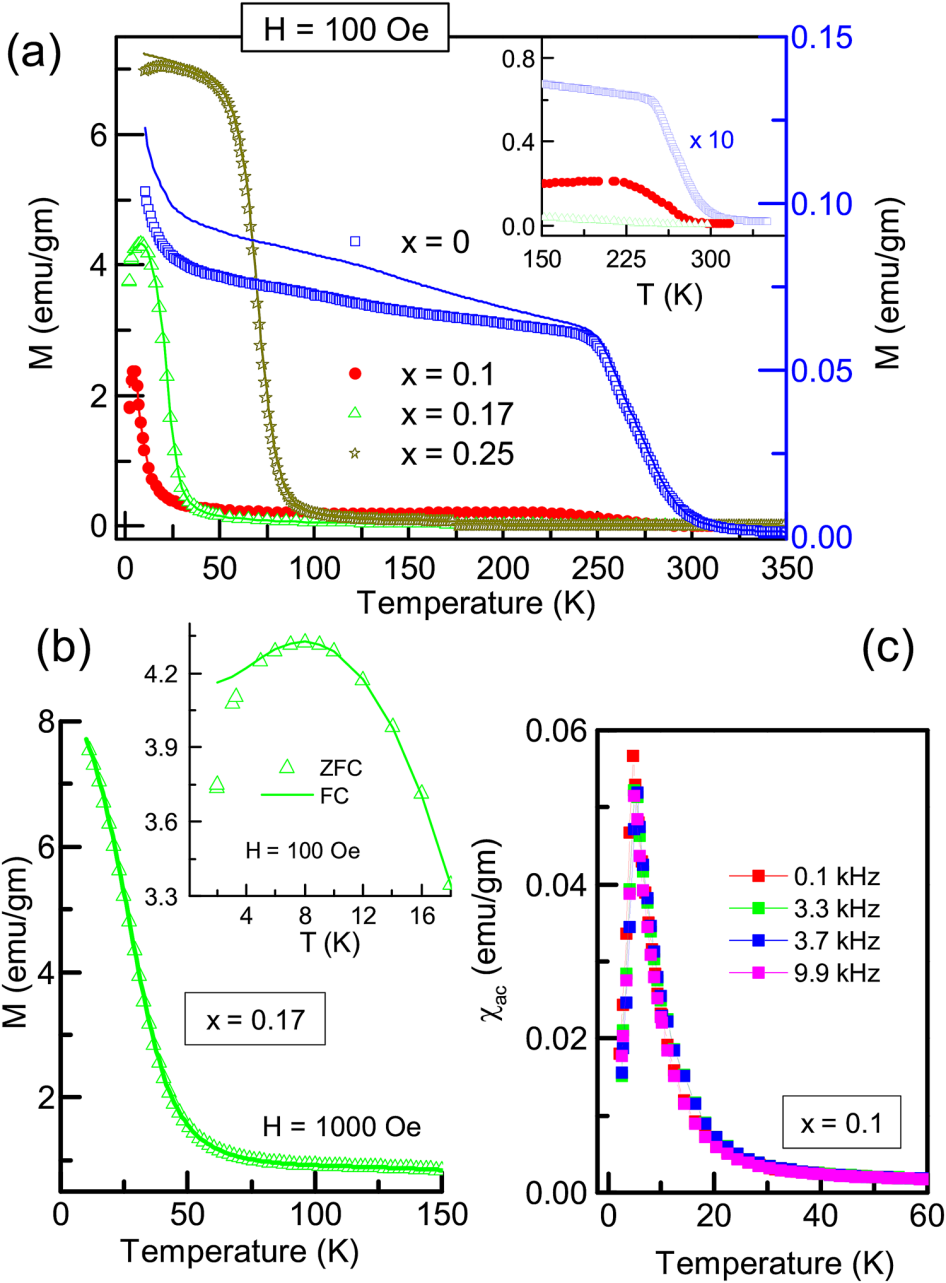
Magnetization vs. temperature measured in ZFC (symbols) and FC (line) protocols: (**a**) measured in an applied field of 100 Oe. The inset here shows systematic suppression of magnetic cluster formation with increasing Cr-substitution. (**b**) measured with an applied field of 0.1 T, and the inset showing ZFC/FC bifurcation for data recorded with H = 100 Oe. (**c**) Low temperature $\chi _{ac}$ measured at different frequencies for a nominal composition.

Magnetization of the ${\text{Fe}}_{2} {\text{Ti}}_{{1 - x}} {\text{Cr}}_{x}$Sn compositions recorded as a function of temperature is shown in Fig. 2a. These plots show M(T) data recorded in zero field cooled (ZFC), and field cooled (FC) measurement protocols. Pristine Fe_2_TiSn exhibits an upward trajectory of M(T) at low temperature, a behaviour typical of its superparamagnetic nature^16^. A previous study shows that magnetic interactions in an otherwise non-magnetic Fe_2_TiSn develop from a small inherent Fe/Ti anti-site disorder^10^. Such interaction leads to the formation of magnetic clusters, identified by a step-like feature in the high-temperature region (270 K) of the M(T) (see inset of Fig. 2a). The clusters so formed, continue to evolve with falling temperature but do not mutually interact to establish any long-range order. The widespread bifurcation between ZFC and FC curves that starts at a much high temperature in Fe_2_TiSn is thus an expected result originating from its inherent Fe/Ti anti-site disorder.

With Cr-substitution, the high-temperature step-like feature weakens and shifts towards lower temperature (inset Fig. 2a) implying a considerable reduction in the magnetic cluster formation, and hence the Fe/Ti(Cr) anti-site disorder. Decrease in temperature sees an abrupt rise in M(T) that marks a transition to the magnetically ordered state. We identify the point of maximum change in the slope of M(T) as the magnetic ordering temperature, $\hbox {T}_p$, and Table 2 summarizes such values. As the temperature decreases further, the ZFC curve shows a downturn along with a small bifurcation from the FC curve. As shown in Fig. 2b, application of a slightly higher magnetic field (0.1 T) is sufficient to suppress these features and M(T) keeps rising with fall in temperature. Such a soft magnetic nature is generally associated with magnetic moments that are easy to reorient upon application of an external magnetic field and can originate from the underlying magneto-crystalline anisotropy and/or competing ferromagnetic (FM)/antiferromagnetic (AFM) interactions.

Based on M(T) alone, it is difficult to comment on the magnetic ground state achieved in the Cr-substituted compositions. The parent Fe_2_TiSn being superparamagnetic at low temperature, the possibility of there being a blocking temperature associated with the ordering of the interacting clusters needs to be scrutinized. A versatile probe to study the dynamics of spin systems is the measurement of ac susceptibility ($\chi _{ac}$) performed using different excitation frequencies. Figure 2c presents such $\chi _{ac}$ plots recorded in the temperature range of 2 to 340 K. No apparent shift in peak position is observed with the changing frequency, ruling out the possibility of any magnetic glassy phase and re-affirms the magnetic transitions detected at $\hbox {T}_p$ to be the long-range magnetic order.

**Figure 3:**
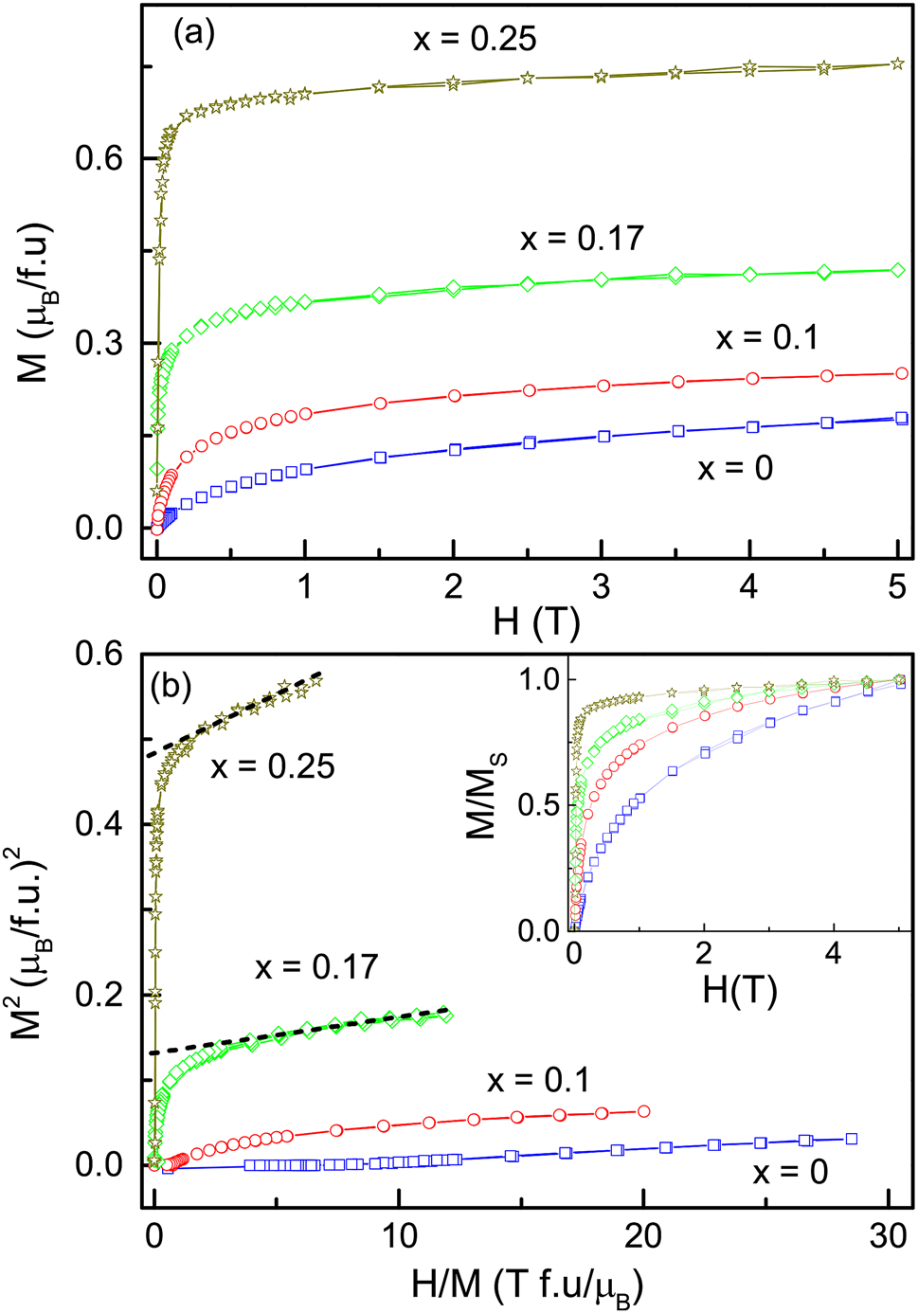
(**a**) M(H) plots for ${\text{Fe}}_{2} {\text{Ti}}_{{1 - x}} {\text{Cr}}_{x}$Sn compositions measured at 5 K. (**b**) Arrot plot extracted for the obtained M(H) curves. Inset presents the normalized M(H) plots indicating the ease of approaching saturation with the increase in Cr concentration.

Next, isothermal magnetization as a function of the applied field, M(H), for all the compositions obtained at 2 K, is shown in Fig. 3. Magnetization does not entirely saturate with ±5 T. However, a smaller value of the applied field is needed for the rapid build-up of magnetization each time the Cr-content increases in the compositions (see inset to Fig. 3b). Further, the Arrot plot in Fig. 3b shows spontaneous magnetization in *x* = 0.17 and 0.25 Cr-substituted compositions as opposed to the linear paramagnetic nature exhibited by Fe_2_TiSn. Summarizing all the observations from magnetization study, it is clear that Cr-substitution in Fe_2_TiSn establishes a long-range magnetic order with underlying competing FM/AFM interactions. The externally applied magnetic field strengthens the favourably oriented interactions and partially reorients the unfavourably oriented ones, resulting in an overall ferrimagnetic ground state in *x* = 0.17 and 0.25 compositions. It becomes pertinent to identify the origin of the underlying FM and AFM interactions as will be discussed in the subsequent sections.

### Heat capacity measurement

**Figure 4:**
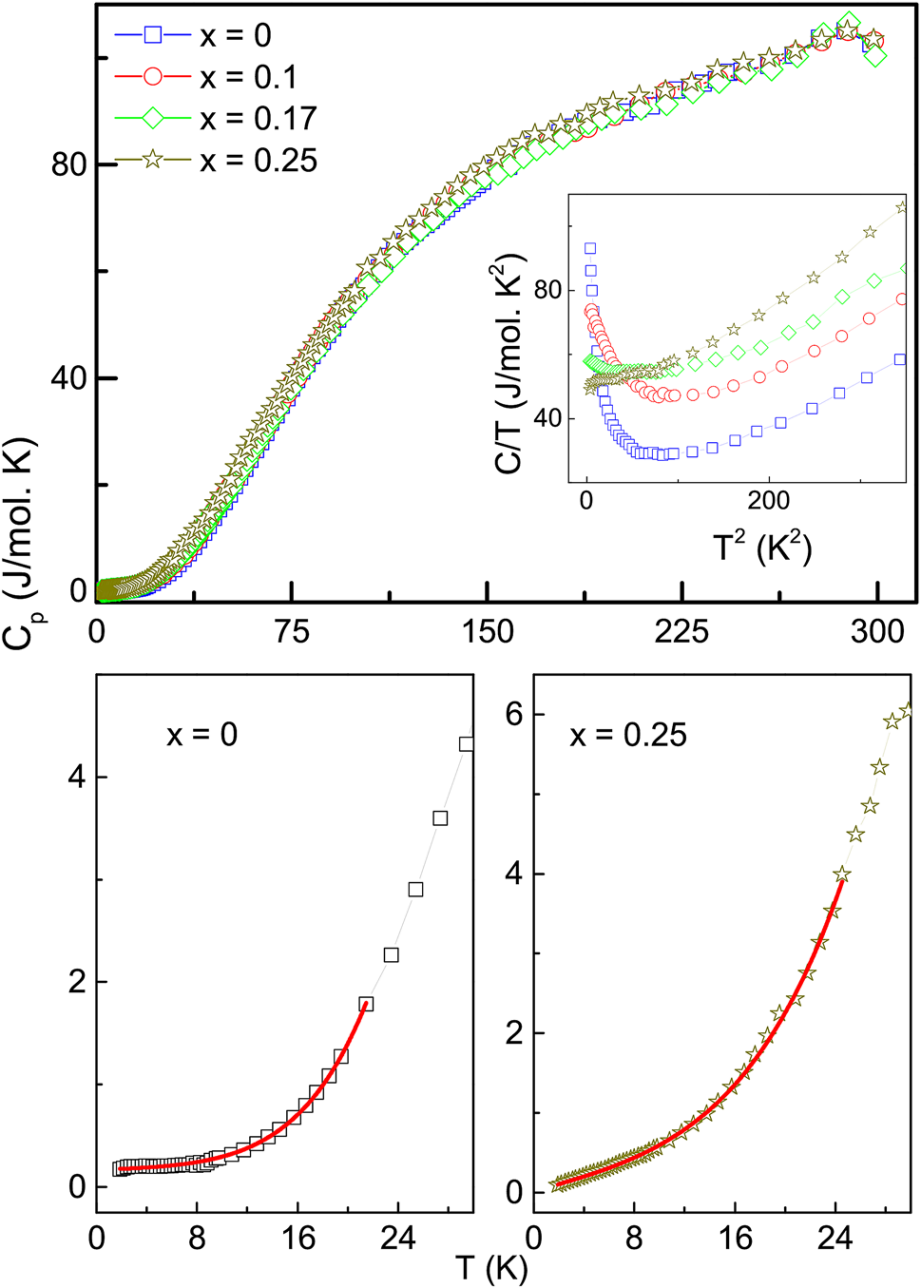
Top panel: Heat capacity measured as a function of temperature for ${\text{Fe}}_{2} {\text{Ti}}_{{1 - x}} {\text{Cr}}_{x}$Sn series. Inset shows the low temperature upturn in C/T. Lower panel: Low temperature heat capacity data fitted with the equation $C(T) = C_0 + \gamma T + \beta T^3 + \delta T^5$ (see text for details). The extracted parameters are summarized in Table 2.

Meanwhile, to further establish that anti-site disorder indeed decreases with Cr-substitution, an alternative probe using thermodynamic measurement was employed. Heat capacity ($C_P$) of ${\text{Fe}}_{2} {\text{Ti}}_{{1 - x}} {\text{Cr}}_{x}$Sn compositions measured in the temperature range 2–300 K are plotted in Fig. 4. The absence of any specific feature near $\hbox {T}_p$ is due to the weak nature of its second-order magnetic transition. The plots of all compositions appear similar to each other when viewed over the entire temperature range. Close inspection of the data in the low-temperature regime bring out significant differences. The specific heat in metallic systems is described as, C(T) = $\gamma$T + $\beta \hbox {T}^{3}$, where γ is Sommerfeld’s coefficient that represents the electronic contribution and β represents the phonon contribution. As presented in the inset of Fig. 4, the plot of C/T vs $\hbox {T}^{2}$ for Fe_2_TiSn shows a clear upturn at low temperature. This upturn diminishes systematically with increasing Cr concentration in the series. As also demonstrated in Fe_2_VAl^17^ and $\hbox {Ru}_{{2}}$NbAl^18^ Heuslers, such an upturn is mainly associated with the presence of magnetic clusters. Generally, the magnetic clusters present in any system rest at lowest energy at low temperature. An external perturbation like temperature or magnetic field alters the cluster’s magnetization vector and it starts oscillating along the direction determined by the magnetic anisotropy, resulting in the form of an anomaly in the low-temperature properties. Such behaviour is indeed observed here for Fe_2_TiSn system that has Fe/Ti anti-site disorder. With increasing Cr-content in $\hbox {Fe}_{{2}}\hbox {Ti}_{1-x}\hbox {Cr}_{{x}}$Sn the up-turn is considerably suppressed, implying a decrease in the formation of magnetic clusters and hence in Fe/Ti(Cr) anti-site disorder.

In such circumstance a modified equation, $C(T) = C_0 + \gamma T + \beta T^3 + \delta T^5$, is often used for description of specific heat^17,19^. The additional terms, $\hbox {C}_{{0}}$ $\sim$ 2$\hbox {k}_{B}N$, accommodates the magnetic cluster formation with the quantity *N* being the number of clusters present in the system. The $\delta T^5$ term includes the extended temperature range beyond $\Theta _{D}$/50 as $\Theta _{D}$, the Debye temperature, has not been separately determined for these compositions^18^. The experimental data fit nicely to this equation in the temperature range (2–25) K, as shown in Fig. 4. As per the extracted parameters listed in Table 2, the value of *N* decreases with increasing Cr-substitution in line with the observation of magnetic measurements discussed in the preceding section. The overall inference here is that the anti-site Fe/Ti(Cr) disorder in Cr-substituted compositions is much less than that found in Fe_2_TiSn. Another significant observation is the rise in the value of γ for compositions with higher Cr content, which indicates stronger electron correlations that may be related to the establishment of long-range magnetic order in this system.

**Table 2:** Various fitting parameters obtained from the analysis of magnetization, heat capacity data and the measured value of residual resistivity ratio (RRR).

Cr	Magnetism		Heat capacity					*RRR* = $\frac{\rho (300)}{\rho (2)}$
conc.	$\hbox {T}_P$	$\hbox {M}_{{s}}$	$\hbox {C}_0$	N$\times 10^9$	$\gamma$	$\beta$	$\delta$	
(x)	K	$\mu _B$	mJ/mol K	per-mol	mJ/mol $\hbox {K}^{2}$	mJ/mol $\hbox {K}^{4}$	mJ/mol $\hbox {K}^{6}$	
0	–	0.104	166.59	1.21	4.89	0.0598	2.05	1.23
0.10	6.0	0.203	141.06	1.02	26.59	0.549	1.997	1.053
0.17	26.4	0.369	58.51	0.425	37.91	0.0969	1.022	0.876
0.25	66.7	0.698	19.46	0.141	43.72	0.1306	0.9863	1.321

### Local structure analysis

**Figure 5:**
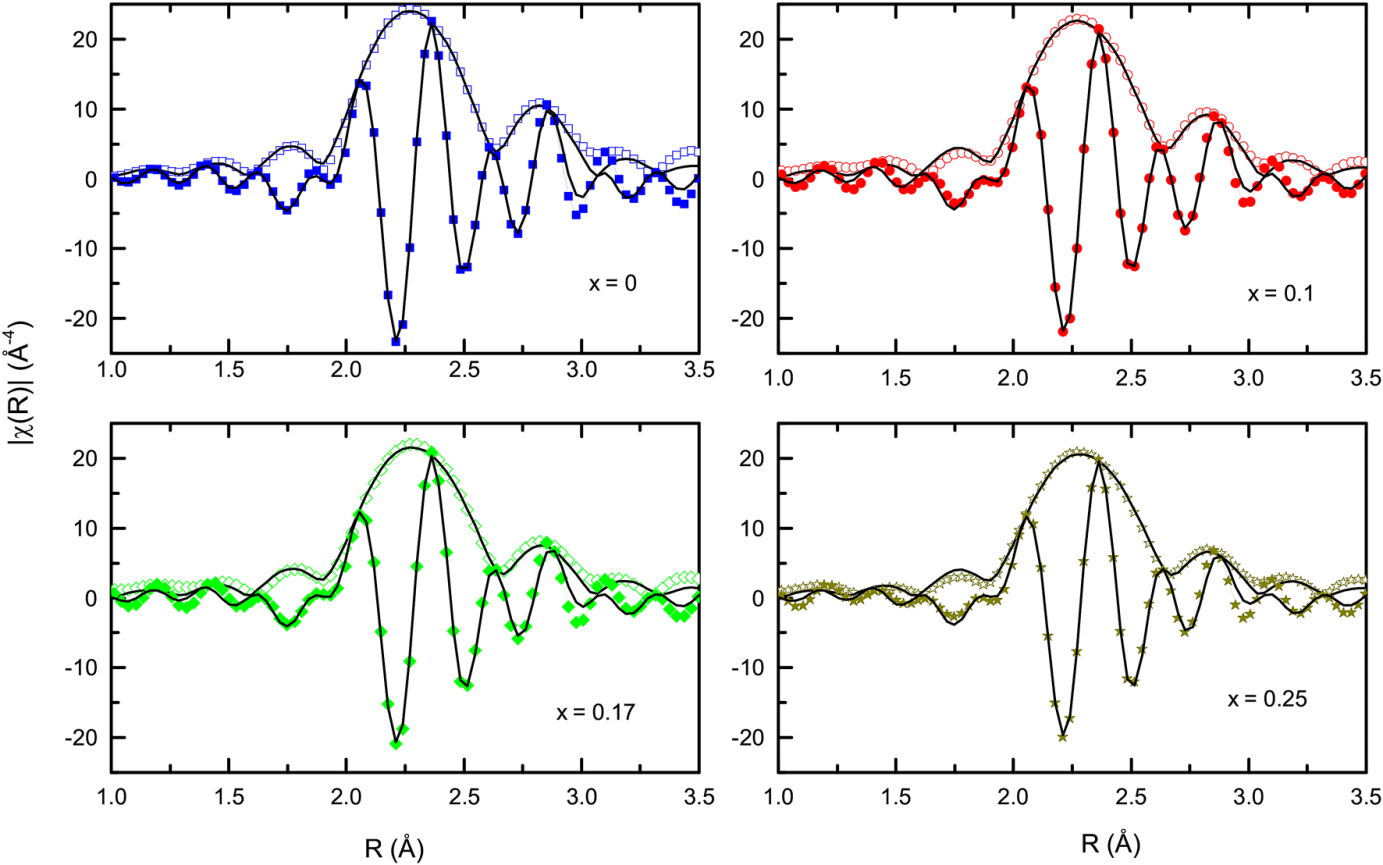
Fitting to the magnitude (hollow symbols) and real component (filled symbols) of Fourier transform of *k*$^{3}$ weighted Fe K-edge EXAFS spectra of ${\text{Fe}}_{2} {\text{Ti}}_{{1 - x}} {\text{Cr}}_{x}$Sn compositions measured at 30 K.

Measurements so far indicate competing FM/ AFM interactions in ${\text{Fe}}_{2} {\text{Ti}}_{{1 - x}} {\text{Cr}}_{x}$Sn compositions, while the origin of these magnetic interactions is not clear. Magnetism in Heusler alloys mainly originates from Ruderman–Kittel–Kasuya–Yosida (RKKY) interactions between magnetic cations mediated via conduction electrons. Hence, the distance between atoms plays a crucial role. Recording the Fe K-edge EXAFS as a function of temperature can help trace any local crystal structural changes that might take place in the Cr-substituted compositions. With the incident X-rays tuned across the K-shell energy of Fe atoms, the variation in the absorption coefficient after transmitting through a given composition is traced. The *k*^3^ weighted data after subtraction of the isolated atom background, plotted as a function of *k* is shown in the supplementary text. These $\chi (k)$ spectra reflect high-quality EXAFS signals up to 15 $\hbox{\AA}^{-1}$ and the data in the range of 3 to 14 $\hbox{\AA}^{-1}$ can be easily Fourier transformed to *R*-space to provide sufficient local structural information. The corresponding $\chi (R)$ plotted as a function of distance *R* from the absorbing atom is shown in Fig. 5.

Atomic arrangements in full Heusler Fe_2_TiSn is such that each Fe atom has 4 Ti and 4 Sn atoms as its nearest neighbours at a distance $d\sim \sqrt{3}\times a/4$ and 6 Fe atoms at $d \sim a/2$. Thus the first prominent peak in Fig. 5 centered around 2.3 Å  contains the contributions from Fe–Ti and Fe–Sn correlations, while the peak at $\sim$ 3.0 Å is mainly due to Fe–Fe correlation. Modeling of the EXAFS data consisted of these three scattering paths and the input from the X-ray diffraction in terms of unit cell parameters values were used as the first guess. Refinement was carried out by varying the atomic distances and thermal mean square factor ($\sigma ^{2}$) until a good fit was achieved in the range of 1.8–3.0 Å in *R* space and 3–14 Å$^{-1}$ in *k* space. The same strategy was adopted for the analysis of the rest of the data obtained at three different temperatures, and for all four compositions. Analysis of the spectra at different temperatures for a given composition does not show any drastic change in the patterns or fitting parameters other than the temperature-related lattice expansion effect. Hence, only the atomic distances and $\sigma ^{2}$ values extracted from the analysis for data recorded at 30 K are listed at Table. 3.

**Table 3:** Atomic distances, thermal mean square variation ($\sigma ^{2}$), coordination number (CN), extracted from the fitting of EXAFS spectra of $\hbox {Fe}_{{2}}\hbox {Ti}_{1-x}\hbox {Cr}_{{x}}$Sn compositions, measured at 30 K.

x	Distance	CN	R (Å)	$\sigma ^{2}$ $(\hbox {AA}^{2}$)
0	Fe–Sn	4	2.62 (1)	0.0035 (05)
	Fe–Ti	4	2.60 (1)	0.0082 (19)
	Fe–Fe	6	3.01 (1)	0.0081 (15)
0.1	Fe–Sn	4	2.62 (1)	0.0038 (04)
	Fe–Ti/Cr	4	2.60 (1)	0.0083 (15)
	Fe–Fe	6	3.02 (1)	0.0090 (13)
0.17	Fe–Sn	4	2.62 (1)	0.0041 (06)
	Fe–Ti/Cr	4	2.59 (1)	0.0084 (18)
	Fe–Fe	6	3.03 (2)	0.0106 (21)
0.25	Fe–Sn	4	2.62 (1)	0.0043 (07)
	Fe–Ti/Cr	4	2.59 (1)	0.0091 (21)
	Fe–Fe	6	3.03 (2)	0.0117 (25)

Despite its cubic symmetry, the Fe–Ti distance in all four compositions turns out to be shorter than the Fe–Sn distance. A probable reason could be the sizeable phase difference between the photoelectrons backscattered from a stronger *d–d* hybridization between Fe and Ti/Cr orbital than the *d–p* hybridization between Fe and Sn orbital. Next, the Fe–Sn and Fe–Fe distances in Fe_2_TiSn are in good agreement with the distances calculated based on cell parameters obtained from XRD profile. As seen earlier, the XRD profile analysis of Cr-substituted compositions indicate a decrease in the lattice constants. However, EXAFS analyses here do not show the corresponding shortening of the Fe–Sn distance. More interestingly, the Fe–Fe distance remains almost constant with increasing Cr–content, albeit with a higher value of $\sigma ^{2}$. An unchanged Fe–Fe distance with substantial thermal mean square variation hints towards presence of a local strain in this bond within the cubic unit cell. As there are no structural transformations taking place in Cr-substituted compositions, the peculiarities observed in the local crystal structure are probably a manifestation of its changing electronic structure.

### Density of states calculation

Electronic-structure-wise, Fe_2_TiSn is a semi-metal with a pseudogap at Fermi level^20^. The DOS in the vicinity of $\hbox {E}_{{F}}$ comprises of Fe 3*d* states, whereas the 5*p* states lie above the Fermi level^21^. Since the Coulomb interaction strength ($\hbox {U}_{{dd}}$) is known to increase with the filling of the *d* band in the early transition metals^22^, it is quite possible that substituting Cr in place of Ti can give rise to a relative localization of *d* orbital in ${\text{Fe}}_{2} {\text{Ti}}_{{1 - x}} {\text{Cr}}_{x}$Sn compositions. Also, as discussed previously in the text, the heat capacity measurements indicate stronger electron correlations in these systems and EXAFS analysis hint towards local lattice distortions that favour a stronger *d–d* hybridization over the *d–p* hybridization. Hence, to further understand the 3*d* hybridization and its impact on the magnetism of ${\text{Fe}}_{2} {\text{Ti}}_{{1 - x}} {\text{Cr}}_{x}$Sn compositions, Heisenberg exchange coupling parameter ($\hbox {J}_{{ij}}$) was calculated using SPRKKR package.

**Figure 6:**
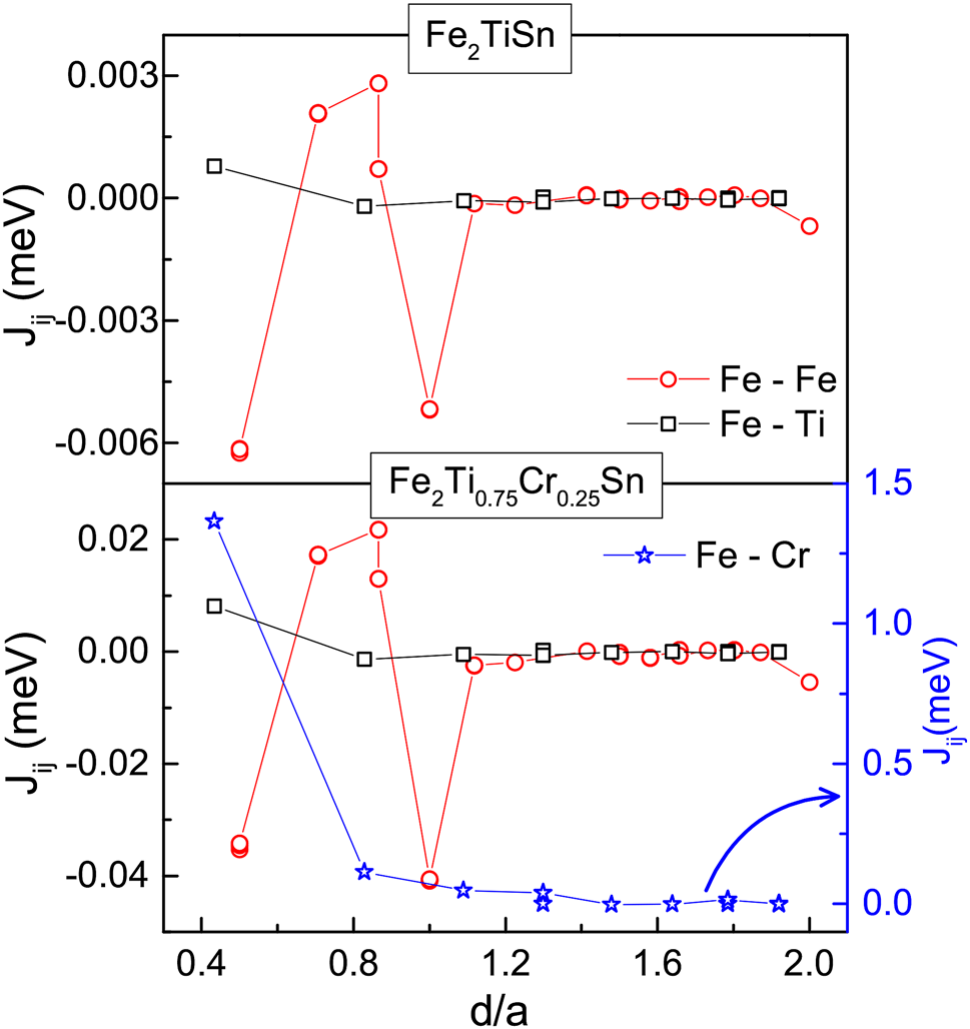
Heisenberg exchange coupling parameter, $J_{ij}$, for Fe–Fe, Fe–Cr and Fe–Ti distances, plotted against the respective bond-distances normalized to the lattice parameter of the corresponding composition.

**Figure 7:**
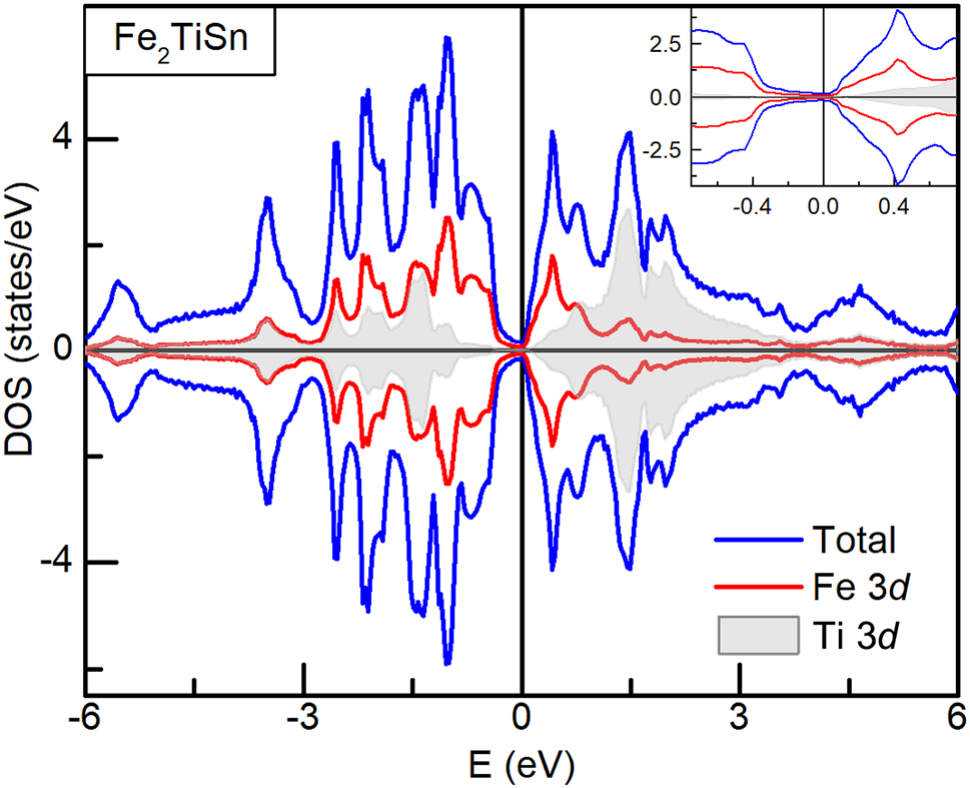
Spin projected total and partial DOS for pristine Fe_2_TiSn composition.

**Figure 8:**
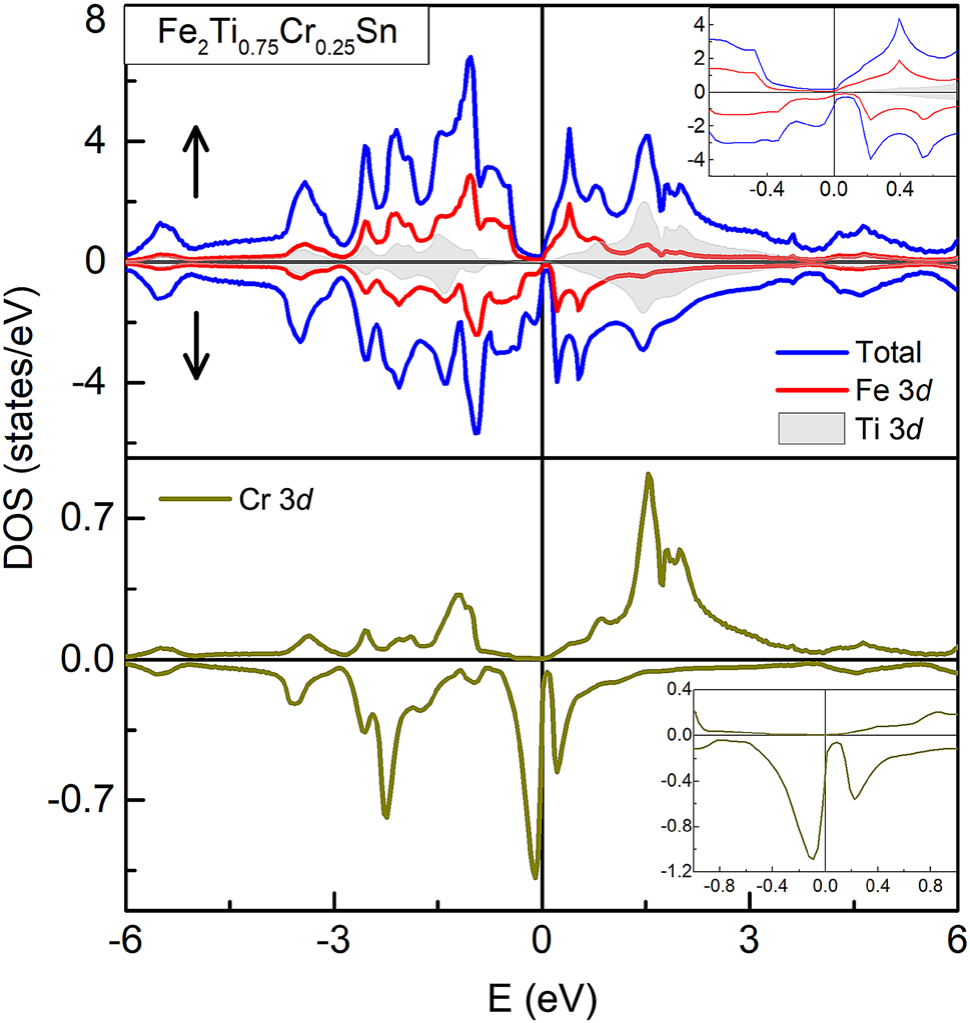
Spin projected total and partial DOS for $\hbox {Fe}_{{2}}\hbox {Ti}_{0.75}\hbox {Cr}_{0.25}$Sn. For clarity pDOS of the Cr *d* state is shown in the lower panel.

Figure 6 presents the obtained exchange coupling parameter for Fe–Fe, Fe–Ti and Fe–Cr distances for both, pristine and *x* = 0.25 composition. The Fe–Fe interaction is oscillatory in nature and dampens with the increase in the separation between the atoms, as per its RKKY character. The Fe–Cr coupling, on the other hand, gives rise to a huge non-oscillatory value of $\hbox {J}_{{ij}}$ ($\sim$ 1.4 meV), supporting a view of localized magnetic moment at Cr site. It is clear from the lower panel of Fig. 6 that Fe–Fe coupling is opposite in sign to Fe–Cr coupling, which forms the basis of competing FM/AFM interactions in the Cr–based compositions. The combination of a localized moment at the Cr site and the RKKY exchange between Fe atoms thus gives rise to the resultant ferrimagnetic ground state in $x \ge$ 0.17 compositions of ${\text{Fe}}_{2} {\text{Ti}}_{{1 - x}} {\text{Cr}}_{x}$Sn. It may seem preemptive at this stage to believe that the $d-d$ hybridization developed in Cr-substituted $\hbox {Fe}_{{2}}$TiSn leads to an ultimate effect of the opening of a gap at $\hbox {E}_{{F}}$ and a consequent half-metallic state. However, the spin-polarized total DOS and orbital projected partial-DOS (pDOS) for Fe_2_TiSn and $\hbox {Fe}_{{2}}\hbox {Ti}_{0.75}\hbox {Cr}_{0.25}$Sn does present such a picture. Plotted at Figs. 7 and 8, are the DOS calculations for Fe_2_TiSn and $\hbox {Fe}_{{2}}\hbox {Ti}_{0.75}\hbox {Cr}_{0.25}$Sn, respectively. In agreement with calculations present in literature, Fe_2_TiSn displays symmetric DOS in both spin channels and a small pseudogap at the Fermi level. With 25% substitution of Cr, the symmetry between majority and minority spin band breaks down with a gap of $\sim$ 120 meV. While the $E_F$ in the majority spin band stays in the gap, the minority spin band gets shifted outside the gap, thus presenting a half-metallic scenario.

The lower panel of Fig. 8 shows a strong peak in the pDOS for Cr *d* band and establishes that the localized magnetic moment is present at Cr site. The calculated magnetic moment of 0.52 $\mu _{B}$/f.u. compares well with the value 0.5 $\mu _{B}$/f.u. estimated from Slater–Pauling empirical rule, but lower than the experimentally observed value of 0.698 $\mu _{B}$/f.u (see Table 2). The discrepancy can arise as the present DOS calculations do not include the electron correlations effect. Inclusion of $\hbox {U}_{{dd}}$ would further strengthen the $d-d$ hybridization and might result in a higher moment estimation. Also, the $\hbox {J}_{{ij}}$ value for Fe–Cr interaction being high, the role of any inherent Fe/Ti(Cr) anti-site disorder though small, cannot be completely ruled out. In Fe_2_TiSn, the extent of such disorder is determined to be $\sim$ 5% and our experimental investigation so far indicates that such percentage is considerably reduced for Cr-compositions. Referring to the Fe–Cr interaction plotted in Fig. 6, the anti-site disordered Cr would be at $d \sim a/2$ with $\hbox {J}_{{ij}}$ value having the same sign and reduced magnitude than the main Fe–Cr interaction. The number of such anti-site disordered pairs being very small, integrating this factor in the resultant magnetization serves as a small correction.

The significant contributor to discrepancy in the experimental and calculated magnetic moment is the externally applied magnetic field. As noticed earlier from the M(T) measurement, an easy re-orientation of magnetic spins takes place for an applied field value of 0.1 T. M(H) curves also display a soft magnetic nature with magnetization that does not fully saturate even at ± 5T. Given that Fe–Cr presents a local moment interaction and much stronger $\hbox {J}_{{ij}}$, it is energetically expensive to reorient such spins. On the other hand, with a much weaker $\hbox {J}_{{ij}}$ the Fe–Fe magnetic clusters bound by RKKY interactions can easily be influenced by the applied field. Besides, the small magnitude of the moment also brings into relevance the orbital moment associated with the constituent atoms of the system, which would have otherwise been a small correction to the total moment value. Thus the magnetism in Cr-substituted compositions poses a complex picture that demands a separate investigation in itself. For the present, the relevant inference is the possibility of the half-metallic state in *x* = 0.25 composition.

### Resistivity measurement

**Figure 9:**
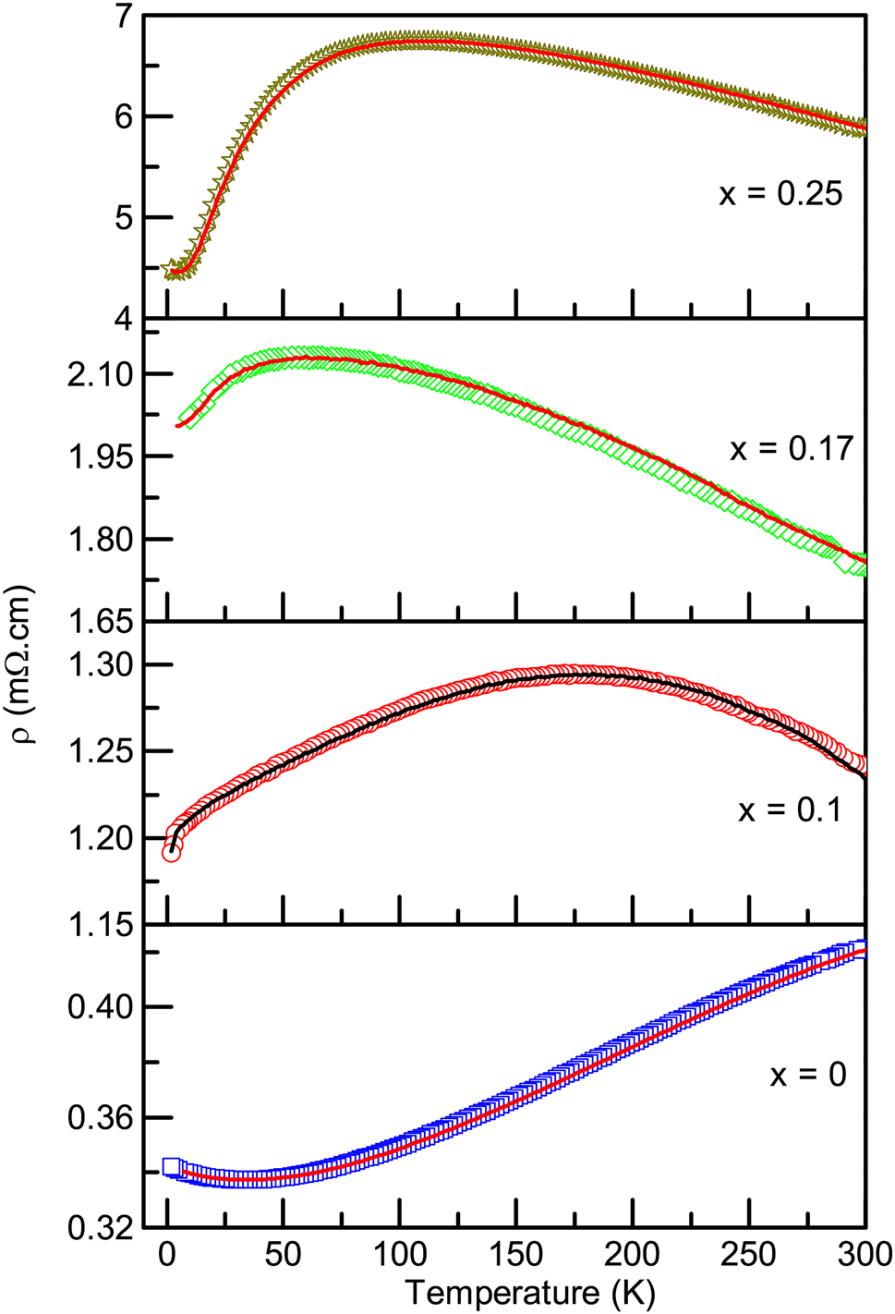
Variation of resistivity with temperature for all prepared compositions of ${\text{Fe}}_{2} {\text{Ti}}_{{1 - x}} {\text{Cr}}_{x}$Sn, recorded during warming (symbol) and cooling (line) cycles.

To substantiate the half-metallic character predicted from the DOS calculations of $\hbox {Fe}_{{2}}\hbox {Ti}_{0.75}\hbox {Cr}_{0.25}$Sn, we carry out the electrical transport measurement for the ${\text{Fe}}_{2} {\text{Ti}}_{{1 - x}} {\text{Cr}}_{x}$Sn series. Fig. 9 presents the temperature-dependent resistivity ($\rho$) measured in the temperature range 2–300 K. $\rho$(T) changes distinctively from a typical metallic character in *x* = 0 to a seemingly insulating nature in ${\text{Fe}}_{2} {\text{Ti}}_{{1 - x}} {\text{Cr}}_{x}$Sn. As there is no hysteresis in the warming/cooling curves of $\rho$(T), the possibility of any first-order structural change does not exist. Therefore, the broad hump in $\rho$(T) should find its origin in the changing electron–magnon scattering due to the complex magnetic order already witnessed in ${\text{Fe}}_{2} {\text{Ti}}_{{1 - x}} {\text{Cr}}_{x}$Sn series. The magnitude of $\rho$ increases sharply by an order of magnitude with the introduction of Cr at Ti-site. $\hbox {Fe}_{{2}}$TiSn shows a metallic value of $\rho (2K) \sim$ 0.34 m$\Omega$cm. With only 10% of Ti being replaced with Cr, the resistivity increases to $\rho (2K) \sim$ 1.1 m$\Omega$cm , and for the maximum substitution of *x* = 0.25, $\rho (2K)$ reaches a value of 4.45 m$\Omega$cm, a value much higher than the typical Ioffe–Regel bad metal limit.

**Figure 10:**
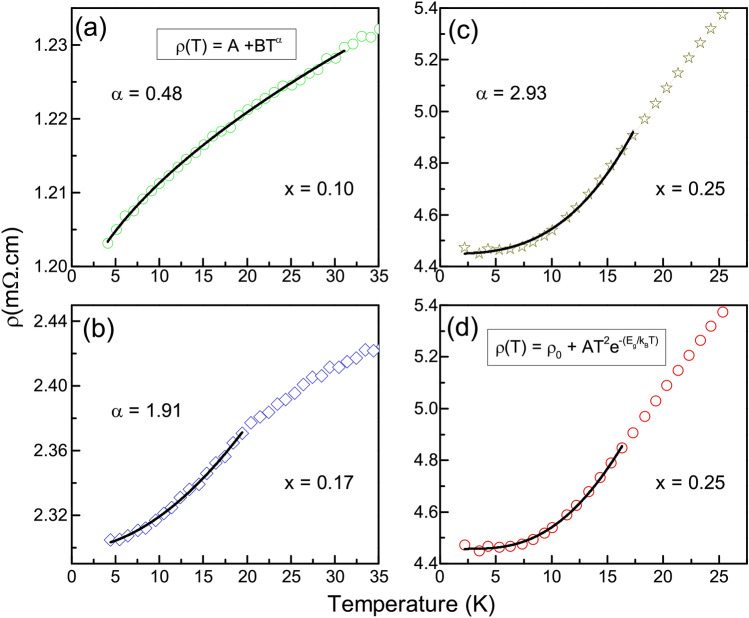
Power law fitted to the low temperature $\rho$(T) plots of Cr-substituted compositions. Line represents the fit to the experimental data represented by symbols.

Electronic transport in the Fe_2_TiSn composition is known to be dominated by holes^10^. Substitution of Cr in place of Ti seems to reduce the total number of charge carrier concentration in the system, thus affecting its overall resistivity. However, such a naive argument is suitable only for $x \le$ 0.17 compositions, where a systematic decrease in the residual resistivity ratio (*RRR*) is observed (see Table 2). A distinctly higher *RRR* in *x* = 0.25 compels the need for a thorough analysis of its low-temperature resistivity. Commonly known half-metallic systems^7,23^ tend to follow an empirical $\hbox {T}^{3}$ dependence of resistivity at low temperature. Accordingly, $\rho$(T) of all the Cr-compositions when fitted with a power law given by $\rho$(T) = A + $\hbox {BT}^{\alpha }$, gives a good fit for different values of exponent $\alpha$, as shown in Fig. 10. For *x* = 0.1, the value of $\alpha$ is close to 0.5, which indicates dominance of weak localization effect^13,24^ in this composition. In case of *x* = 0.17, $\alpha$ is close to 2, which indicates a normal metallic transport dominated by electron–electron correlation, and for *x* = 0.25, $\alpha$ indeed turns out to be close to 3, in congruence with the other half-metallic systems.

A direct exploration of the half-metallic gap requires probes that can be spin polarized and techniques like the point contact Andreev reflection (PCAR) spectroscopy or angle-resolved Photoemission spectroscopy may be the better alternatives. A simple four-probe resistivity measurement such as the one presented here cannot provide the measure for half-metallic gap. Besides, in real systems and at non-zero temperatures, there will be a finite effective minority DOS at $\hbox {E}_F$ due to inherent factors like atomic disorders, spin-fluctuations in the localized *d* orbital etc. However, indirect signatures of half-metallicity can be traced from the resistivity measurement. For example, the electron–magnon scattering is drastically dampened in half-metallic systems. Electron–magnon being a spin flip scattering process, needs the presence of both spins to complete a full spin wave. The inaccessibility of minority spin states being the requisite for a half-metallic state, electron–magnon scattering is impossible at low temperature in such systems. According to Watts et al.^25^ the decrease in electron–magnon scattering is exponential and the resistivity can be described by the empirical rule, $\rho$(T) = $\rho _{0}$ + $\hbox {AT}^{2}$ $\exp {(-\frac{E_{g}}{k_{B}T})}$. Such fitting has previously been attempted for several other half metallic systems like $\hbox {CrO}_2$^3^, Fe_2_CoSi^7^. In absence of any other theoretical model for estimation of the half-metallic gap, we adopt such a fitting procedure for *x* = 0.25 and obtain a value of $\sim$ 14.3 meV. The fitting is presented at Fig. 10d. It may be noted that $E_g$ represents the factor by which a single magnon scattering process would be suppressed as T $\rightarrow$ 0. The temperature scale associated with $\hbox {E}_g$ ($\sim$ 160 K) implies that the spin polarization persists on a local scale much above the magnetic ordering temperature, $\hbox {T}_p$.

**Figure 11:**
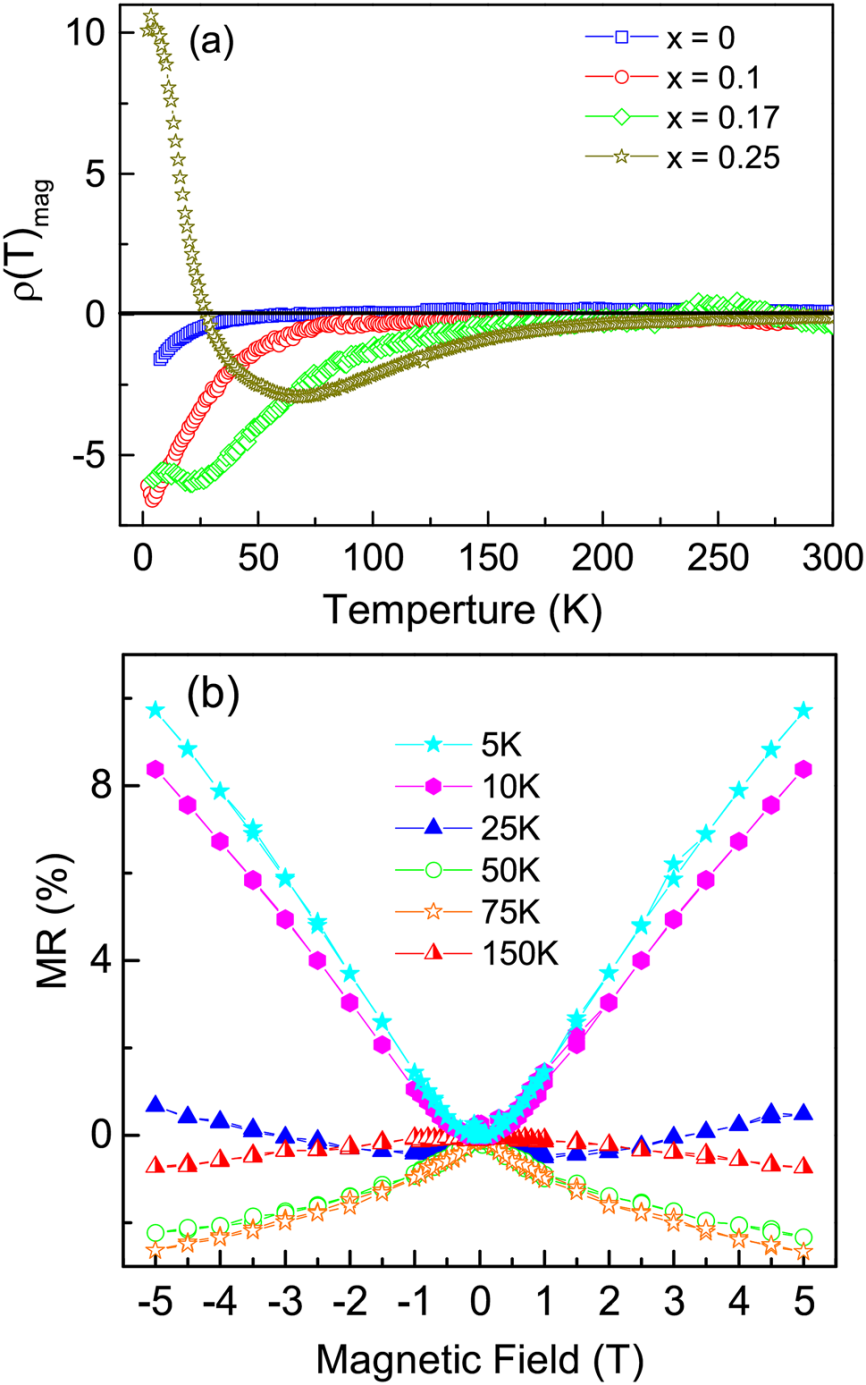
(**a**) Magnetoresistance measured as the function of temperature in presence of a fixed 5 T magnetic field for all $\hbox {Fe}_{{2}}\hbox {Ti}_{1-x}$ $\hbox {Cr}_{{x}}$Sn compositions. (**b**) Isothermal magnetoresistance measured as the function of applied field, for $\hbox {Fe}_{{2}}\hbox {Ti}_{0.75}\hbox {Cr}_{0.25}$Sn.

Characteristic anomalies associated with half-metallic ground state of *x* = 0.25 can also be found in its magnetic field dependent electrical transport measurement. As depicted in Fig. 11a, decrease in $\rho$(T)$_{mag}$ = $\frac{\rho (T)_{5T} - \rho (T)}{\rho (T)}$ $\times$ 100 is observed with falling temperature, that reaches a highest negative $\rho$(T)$_{mag}$ value at $\hbox {T}_p$. With further decrease in temperature, $\rho$(T)$_{mag}$ changes direction and rises to a relatively high positive value. $\rho$(T)$_{mag}$ crosses zero at $\sim$ 25 K. Alternative measurement of the isothermal magnetoresistance, $\rho$(H), at fixed temperatures shows equally fascinating results. A considerably high and positive value of MR (MR = $\frac{\rho _H - \rho _0}{\rho _0}\times$ 100) is obtained below 25 K, as displayed in Fig. 11b. What is exceptional here is the linear variation of MR with the applied magnetic field. Generally, an ordinary ferromagnetic material displays negative MR as the application of the magnetic field decreases the entropy of the system in the Fermi liquid-like state. On the other hand, a positive value of MR is due to Lorentz force on resistivity in the presence of magnetic field^26^. In either case, MR follows a quadratic magnetic field dependence that saturates at sufficiently high field. Interestingly, the MR observed for *x* = 0.25 is a large positive value that changes linearly with the applied field. Due to freezing out of electron–magnon scattering in the half-metallic phase, the only process that stays relevant at low temperature is the electron–defect scattering. This electron–defect scattering increases upon application of the magnetic field, which give rise to a positive linear magnetoresistance^6^. Thus the half-metallic nature of $\hbox {Fe}_{{2}}\hbox {Ti}_{0.75}\hbox {Cr}_{0.25}$Sn finds expression in every physical property measured here.

## Discussion

In conclusion, we have investigated the $\hbox {Fe}_{{2}}\hbox {Ti}_{1-x}$ $\hbox {Cr}_{{x}}$Sn compositions in search for the half-metallic ground state. Our work was motivated by the theoretical prediction of the half-metallic ground state in Fe_2_CrSn. However, through our crystal structure study using synchrotron XRD, we show that pure Fe_2_CrSn does not crystallize in Heusler structure. Though, it is possible to partially replace up to 25% of the Ti atoms with Cr in another stable Heusler composition, Fe_2_TiSn. The heat capacity and magnetization measurements provide sufficient evidence for a decrease in the anti-site disorder between Fe/Ti(Cr) sites. Existence of such disorder is known to cause a superparamagnetic phase in non-magnetic Fe_2_TiSn. Magnetization measurements carried out on $\hbox {Fe}_{{2}}\hbox {Ti}_{1-x}$ $\hbox {Cr}_{{x}}$Sn compositions show presence of competing FM/ AFM magnetic interactions. At 25% Cr substitution the resultant magnetization appears to be a ferrimagnet. The heat capacity measurements provide evidence for the presence of strong electron correlations. Fe K-edge EXAFS analysis provides complementary information related to Fe–Ti, Fe–Sn and Fe–Fe distances, that help understand the RKKY interactions in this system. The decrease in 3*d*–5*p* hybridization which enhances the localization of Cr 3*d* states, leads to the opening of the gap in one of the spin channels as seen from our DOS and Heisenberg exchange coupling parameter calculations. A consistent picture from the experimental studies and calculations emerges for the ${\text{Fe}}_{2} {\text{Ti}}_{{1 - x}} {\text{Cr}}_{x}$Sn series. Linear positive magnetoresistance at low temperature confirms the half-metallic ground state in $\hbox {Fe}_{{2}}\hbox {Ti}_{0.75}\hbox {Cr}_{0.25}$Sn. Analysis of the low-temperature resistivity estimates the spin-flip magnon gap to be of the order of $\sim$ 14.3 meV.

## Methods

$\hbox {Fe}_{{2}}\hbox {Ti}_{1-x}\hbox {Cr}_{{x}}$Sn compositions were prepared by arc melting of stoichiometric quantities of constituent elements (purity $\ge$ 99.999%) under Ar atmosphere. The ingot was melted several times to ensure homogeneity and was annealed in an evacuated quartz tube at 1073 K for 72 h before quenching into ice water. The final compositions were analyzed using Supra55 Zeiss field-emission scanning electron microscope equipped with Oxford instruments AZtec energy dispersive microanalysis system. The elemental ratio in each composition finds a match with the starting stoichiometry. The phase purity and homogeneity of the prepared compositions were examined using synchrotron-based powder X-ray diffraction technique. Samples in finely powdered form were used for the measurement at the Extreme Conditions Angle Dispersive/Energy dispersive X-ray diffraction (AD/ED-XRD) beamline (BL-11) at INDUS-2 synchrotron source, Raja Ramanna Centre for Advanced Technology (RRCAT), Indore, India. During measurement, the sample powders loaded in a capillary, are rotated at  150 rpm to reduce any orientation effects. Desired wavelength ($\lambda$ = 0.612 Å) for AD-XRD diffraction experiments was selected from the bending magnet using a Si(111) channel-cut monochromator.

$\hbox {Fe}_{{2}}\hbox {Ti}_{1-x}\hbox {Cr}_{{x}}$Sn compositions were characterized by recording its temperature and magnetic field dependent electrical transport, heat capacity and magnetic properties. Quantum Design Inc. Physical Properties Measurement System (PPMS) and Superconducting Quantum Interference Device magnetometry with Vibrating Sample Magnetometer (SQUID-VSM) were extensively used for such measurements. EXAFS was carried out at P65 undulator beamline, PETRA-III DESY, Hamburg, Germany. Here, a fixed-exit Si(111) double-crystal is used as a monochromator and Si plane mirror is used for harmonic rejection. The X-ray intensity is measured using two ionization chambers, before and after the sample stage. We recorded the spectra in transmission mode at the Fe K–edge, and at various sample temperatures. A thin foil of Fe-metal placed after the second detector was used as a standard for EXAFS signal and to calibrate the monochromator edge. Temperature variation at the sample stage was achieved using an Oxford Instruments liquid Helium flow cryostat. X-rays were tuned to the energy interval of 6800–8000 eV and made incident on the sample. Open-source data analysis packages like FEFFIT program^27^ that uses ATOMS and FEFF9 program^28^ as the basis for the calculation of theoretical fitting standards, were used.

Spin-polarized-relativistic Korringa–Kohn–Rostoker method (SPR–KKR) was used to calculate the Heisenberg exchange coupling parameters($\hbox {J}_{{ij}}$) and density of states(DOS), as implemented in SPRKKR program package^29^. A *k* point mesh of 36 × 36 × 36 was implemented in the Brillouin zone for self-consistent calculation. Angular momentum expansion for each atom was taken such that *l*$_{max}$ = 3. Generalized gradient approximation (GGA) was used for exchange-correlation functional. For the calculation of DOS a finer mesh of 49 × 49 × 49 was used, and in all calculations the energy convergence criterion was were set to 10^−6^ Ry. Other parameters that were used for the calculation of DOS are NKTAB = 2300, NE = 450, ImE = 0.001 Ry.

The $\hbox {J}_{{ij}}$ calculation is based on the method proposed by Lichtenstein et al.^30^ where magnetic force theorem is used to calculate the $\hbox {J}_{{ij}}$ following the equation, $J_{ij} = \frac{1}{4\pi }\int _{E_{F}}dE Im Tr_{L}(\Delta _{i}\tau ^{ij}_{\uparrow }\Delta _{j}\tau ^{ij}_{\downarrow })$ Here, $\tau$ is the scattering path operator, $\Delta$ is the difference between the inverse single site matrices of up and down spin and $\hbox {Tr}_{{L}}$ is the trace of scattering matrices^31^. The calculation of $\hbox {J}_{{ij}}$ has been done with the cluster radius of 2*a*, where *a* is the lattice parameter. For calculation of $\hbox {J}_{{ij}}$ other parameters, like NKTAB = 2500, NE = 500, ImE = 0.001 Ry were used.

## Supplementary Information

Supplementary Information
