# Supplementary material for: Half metallicity in Cr substituted Fe2TiSn
Source: Sci Rep. 2021 Jan 12;11:524. doi: 10.1038/s41598-020-79895-7 (PMC7803981; doi:10.1038/s41598-020-79895-7)
Supplement: Supplementary file 1 — Supplementary Information [file 41598_2020_79895_MOESM1_ESM.pdf]

Supplementary Text:

Half metallicity in Cr substituted Fe_2TiSn

S. Chaudhuri[1], D. Salas[2], V. Srihari[3], E. Welter[4], I. Karaman[2], and P. A. Bhobe[1,*]

[1]Department of Physics, Indian Institute of Technology Indore, Khandwa Road, Simrol, Indore 453552, India
[2]Department of Materials Science and Engineering, Texas A&M University, College Station, TX, 77843, USA
[3]High Pressure and Synchrotron Radiation Physics Division, Bhabha Atomic Research Centre, Mumbai 400 085, India
[4]Deutsches Elektronen-Synchrotron – A Research Centre of the Helmholtz Association, Notkestraße 85, D 22607 Hamburg, Germany
*pbhobe@iiti.ac.in

Elemental analysis using Energy Dispersive X–Ray (EDX) Spectroscopy. Figures in parenthesis represent the error in last digit.

Cr conc. (x)	Atomic % Fe	Ti	Cr	Sn	Stoichiometry
0	49.35(20)	24.65(72)	–	26.01(78)	$Fe_{1.97}Ti_{0.99}Sn_{1.04}$
0.10	49.75(58)	22.37(24)	2.55(02)	25.33(35)	$Fe_{1.99}Ti_{0.89}Cr_{0.10}Sn_{1.01}$
0.17	49.53(10)	20.63(06)	4.17(05)	25.66(09)	$Fe_{1.98}Ti_{0.83}Cr_{0.17}Sn_{1.02}$
0.25	49.77(36)	18.74(20)	6.07(21)	25.47(34)	$Fe_{1.99}Ti_{0.74}Cr_{0.25}Sn_{1.02}$

Table 1: Elemental analysis using EDX.

XAFS analysis of $Fe_2Ti_{0.75}Cr_{0.25}Sn$

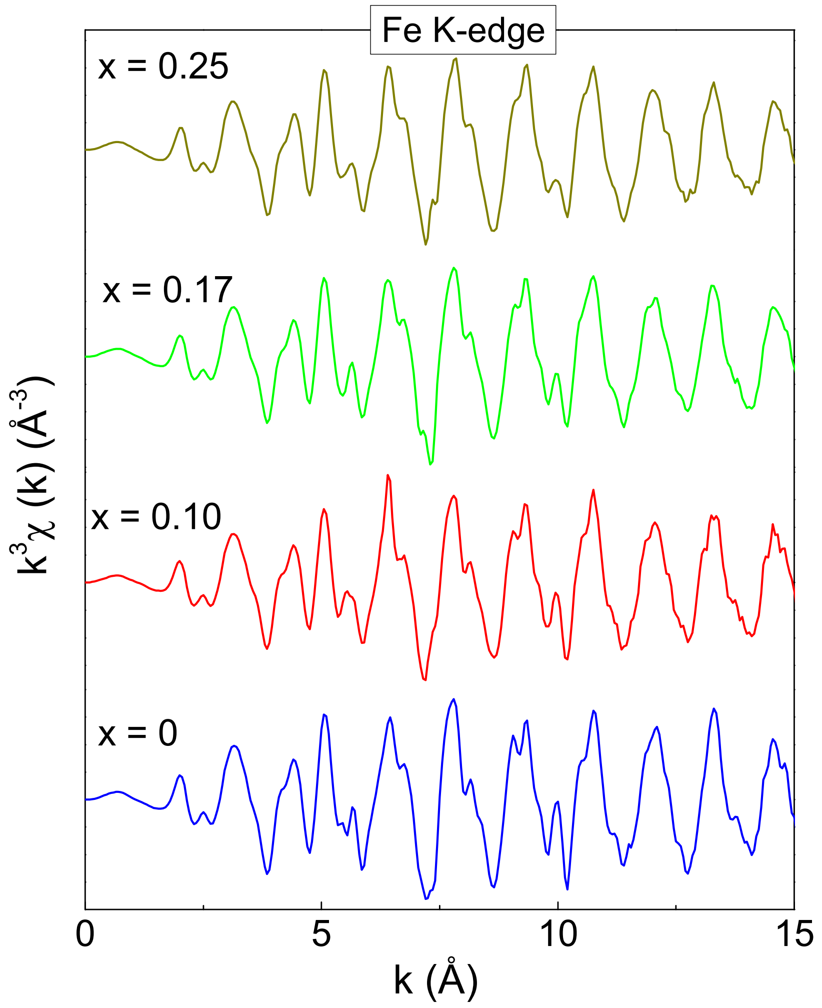

Figure 1: k^3 weighted Fe K-edge EXAFS patterns recorded at 30 K for $Fe_2Ti_{1-x}Cr_xSn$ compositions.

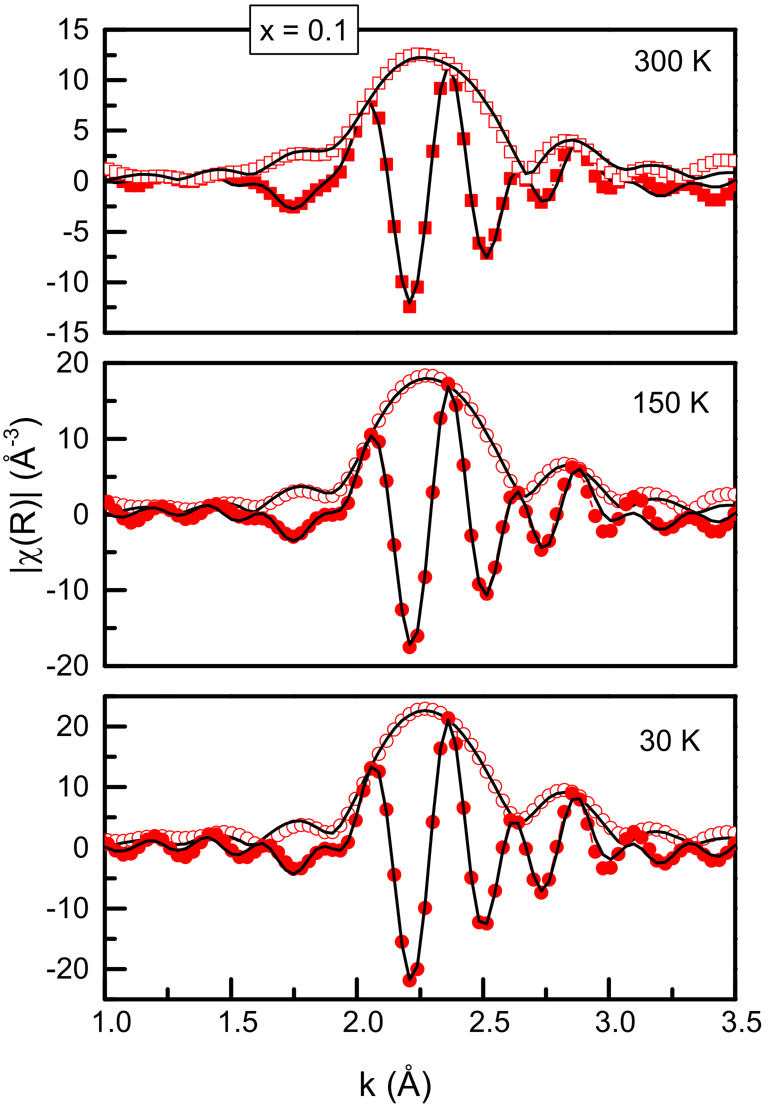

Figure 2: Fitting of the magnitude (hollow spheres) and real component (filled spheres) of Fourier transform of k^3 weighted Fe K-edge EXAFS spectra for $x = 0.1$, measured at different temperatures.
